# Mapping QTL associated with resistance to *Pseudomonas syringae* pv. *actinidiae* in kiwifruit (*Actinidia chinensis* var. *chinensis*)

**DOI:** 10.3389/fpls.2023.1255506

**Published:** 2024-03-26

**Authors:** Casey Flay, V. Vaughan Symonds, Roy Storey, Marcus Davy, Paul Datson

**Affiliations:** ^1^ School of Natural Sciences, Massey University, Palmerston North, New Zealand; ^2^ The New Zealand Institute for Plant and Food Research Limited, Te Puke, New Zealand; ^3^ Kiwifruit Breeding Centre, Te Puke, New Zealand

**Keywords:** selection mapping, WGS, pool sequencing, QTLseqR, bulk segregant analysis

## Abstract

*Pseudomonas syringae* pv. *actinidiae* (Psa) is a bacterial pathogen of kiwifruit. This pathogen causes leaf-spotting, cane dieback, wilting, cankers (lesions), and in severe cases, plant death. Families of diploid *A. chinensis* seedlings grown in the field show a range of susceptibilities to the disease with up to 100% of seedlings in some families succumbing to Psa. But the effect of selection for field resistance to Psa on the alleles that remain in surviving seedlings has not been assessed. The objective of this work was to analyse, the effect of plant removal from Psa on the allele frequency of an incomplete-factorial-cross population. This population was founded using a range of genotypically distinct diploid *A. chinensis* var. *chinensis* parents to make 28 F_1_ families. However, because of the diversity of these families, low numbers of surviving individuals, and a lack of samples from dead individuals, standard QTL mapping approaches were unlikely to yield good results. Instead, a modified bulk segregant analysis (BSA) overcame these drawbacks while reducing the costs of sampling and sample processing, and the complexity of data analysis. Because the method was modified, part one of this work was used to determine the signal strength required for a QTL to be detected with BSA. Once QTL detection accuracy was known, part two of this work analysed the 28 families from the incomplete-factorial-cross population that had multiple individuals removed due to Psa infection. Each family was assigned to one of eight bulks based on a single parent that contributed to the families. DNA was extracted in bulk by grinding sampled leaf discs together before DNA extraction. Each sample bulk was compared against a bulk made up of WGS data from the parents contributing to the sample bulk. The deviation in allele frequency from the expected allele frequency within surviving populations using the modified BSA method was able to identify 11 QTLs for Psa that were present in at least two analyses. The identification of these Psa resistance QTL will enable marker development to selectively breed for resistance to Psa in future kiwifruit breeding programs.

## Introduction

Many cultivars of kiwifruit are devastated by the bacterial pathogen Psa (*Pseudomonas syringae* pv. *actinidiae* biovar 3), also known as the virulent form of Psa (Psa-V)(Everett et al., 2011; McCann et al., 2013; Dwiartama, 2017). This disease is particularly destructive to *A. chinensis* var. *chinensis* (Datson et al., 2015) genotypes, but also affects *A. chinensis* var. *deliciosa* (Takikawa et al., 1989). It has been reported that Psa spread from Asia, where up to four biovars were present (Koh et al., 1994). Each of the non-virulent biovars had different pathogenesis and molecular characteristics on different kiwifruit genotypes, but they did not cause the pathogenesis observed in the virulent Psa-biovar-3. This biovar, first reported in 2010 in New Zealand, is now widespread in the north island of the country, where kiwifruit is widely cultivated. Infected plants show symptoms such as leaf-spotting, cane dieback, wilting, or oozing a clear, brown or white liquid in spring or autumn from cankers (lesions). In highly susceptible genotypes, these symptoms occur on multiple canes leading to whole vine death. On more resistant genotypes, symptoms can involve flower bud browning, bud drop, flower wilting, and leaf spotting (Everett et al., 2011). To manage the outbreak, regulations were established by the national agency, Kiwifruit Vine Health (KVH), which required the removal of plants with severe symptoms such as cankers, or multiple dead canes. with severe symptoms such as cankers, or multiple dead canes, were removed.During the initial outbreak in 2011, the leading yellow-fleshed *A. chinensis* var. *chinensis* cultivar in New Zealand, named ‘Hort16A’, along with its pollenisers, were particularly susceptible to Psa. This susceptibility led to a significant decrease in gold kiwifruit production (Dwiartama, 2017). To address this decline, a gold-fleshed *A. chinensis* var. *chinensis* cultivar with the PVR name ‘Zesy002’ (fruit marketed as Zespri™ SunGold) was utilised to replace gold fruit production that was previously reliant on ‘Hort16A’ (Everett et al., 2011; Dwiartama, 2017). This replacement cultivar exhibits greater resistance to Psa.

As Psa is such a ubiquitous and damaging pathogen, incorporation of resistance to Psa is required for any kiwifruit exposed to field conditions. Current breeding programmes that are based on crossing Psa-resistant families retain moderate resistance. However, due to the highly polygenic nature of resistance to Psa, strong resistance has not yet been achieved in the gold-fleshed *A. chinensis* var. *chinensis* (Tahir et al., 2018; Tahir et al., 2019). QTLs for resistance to Psa have been identified in two families of *A. chinensis* resulting from a resistant by susceptible cross, but identifying these QTL required large replicated trials from a single family and detailed phenotyping (Tahir et al., 2019). The phenotyping requirement, and the requirement of large, replicated families to generate QTL for resistance to Psa, could be overcome by identifying alleles remaining in breeding populations after the selective sweep caused by severe Psa infection.

When Psa spread within kiwifruit families established at Plant & Food Research, Kerikeri, New Zealand, notable differences in the extent of seedling removal emerged among these families due to Psa (personal communication Paul Datson).

The selective sweep caused by Psa presented a chance to investigate allele loci that persisted within the diverse families that made up this population. This investigation was carried out by using a technique called bulk segregant analysis (BSA), which aimed to gain insights into the genetic makeup related to resistance and susceptibility to Psa. BSA operates by assessing alterations in allele frequencies between populations that have segregated due to the pressures of selection, resembling a selection map (Michelmore et al., 1991; Wisser et al., 2008; Magwene et al., 2011; Li and Xu, 2022; Shen and Messer, 2022). The BSA technique has been used for the detection of QTL for target traits in various species, including dwarfing in watermelon (Dong et al., 2018), cotyledon colour in soybean (Song et al., 2017), cold resistance in rice (Sun et al., 2018), resistance to ascochyta blight in chickpea (Deokar et al., 2019), and kernel length-width ratio in wheat (Xin et al., 2020). A typical BSA investigates loci that differ between sample bulks segregating for a trait of interest, combining ideas from linkage mapping and GWAS (Michelmore et al., 1991; Li and Xu, 2022; Shen and Messer, 2022). Like classical linkage mapping, most BSA trials are designed using two parents with different phenotypes. The two parents are crossed to generate an F_1_ population which is back-crossed or interbred for several generations to generate sufficient recombination to break up linkage from parents (Michelmore et al., 1991). Individuals from the last generation are selected to form two bulks that segregate for the phenotype of interest. Thus, alleles affecting the target phenotype should show a significant difference in frequency between the two bulks, while unselected alleles should remain in both bulks at similar frequencies (Michelmore et al., 1991; Shen and Messer, 2022). Diverging from the typical BSA, bulks can be analysed with BSA directly from F_1_ populations (Dai et al., 2018; Guan et al., 2019). Similarly, selection mapping approaches compare a shift in allele frequency between two bulks created from samples of the population before and after a selection event altered the population’s allele frequency (Wisser et al., 2008; Johnsson, 2018). The DNA that contributes to each of the bulks in BSA and selection mapping approaches is typically quantified for each individual, and an equivalent amount of DNA added to the bulk from each individual (Song et al., 2017; Dong et al., 2018; Munjal et al., 2018; Wang et al., 2021). While this approach ensures that a precise quantity of DNA is added from each individual, tracking samples and extracting DNA from individuals is costly and time-consuming. Moreover, both methods will include signal noise from a shift in allele frequency not caused by the target selection pressure. These unintended shifts in allele frequency can be caused by the genetics of the founding parents (James, 1970; Conolly et al., 2008; Chen et al., 2019).

An alternative approach to bulking DNA samples after extraction would be to bulk leaves of different individuals prior to DNA extraction. This approach would simplify sampling and reduce the cost and workload involved with DNA extraction by extracting DNA directly from a bulk of leaf samples. To help standardise the DNA contribution from each sample, the leaf sample growth stage and the amount of leaf material would need to be kept consistent. Samples from each individual then could be ground together for DNA extraction as a bulk. However, this approach precludes a precise balance of each individual’s DNA contribution to the pool and may introduce greater variance into the bulks; therefore individuals that potentially contribute a greater amount of DNA would make a greater contribution to the allele frequencies than others with less DNA extracted. This may decrease the power to identify allelic differences between bulks and thus QTL. Prior to applying such a modified method to an experimental population, a test of the approach to detect selection at a known site would need to be performed to determine its accuracy.

Testing the level of precision of the modified method of bulk sampling would require a population segregating for a simple control trait that is determined by a single well-characterised locus, and ideally with low interaction between the gene and the environment. To this end, within the dioecious *A. chinensis*, plant sex is a suitable trait as it is easy to phenotype and it is controlled by a single well-characterised dominant gene that is not affected by the environment (Akagi et al., 2018). This kiwifruit sex gene, named *Shy Girl* (*SyGl*) exists on the male Y chromosome and suppresses flower feminisation, producing males in plants possessing it (Akagi et al., 2018). It was assumed that if a shift in allele frequency could be detected using these methods in the monogenic *Shy Girl* gene, polygenic loci of strong influence on the population would also be able to be detected.

This study aimed first to test whether pooling leaves from multiple individuals prior to DNA extraction enables a BSA to be effectively carried out on the resulting DNA pool. This was done using a series of bulked pools that varied in the ratio of male and female *A. chinensis* individuals that contributed to the pools and investigated whether the QTL for plant sex could be identified on chromosome 25. Part two of this work aimed to identify any changes in allele frequency between bulks of sample pools of seedlings that had survived in the field following a Psa selective sweep and a bioinformatically generated bulk of data from parents contributing to each sample bulk. Regions of the genomes where alleles have a greater sample depth in the WGS of sample bulk data than expected from their parental bulk of data should highlight the regions of the genome under strong selection from Psa.

## Materials and methods

### Population

**Figure 1:**
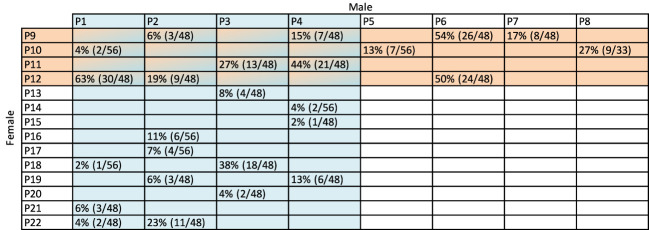
The crossing structure of 25 families with surviving individuals from the 12x18 population. For part two of this work, eight bulks were made by sampling leaves from all surviving plants within families which shared the parent indicated in the blue columns and salmon rows. Female parents (P9-P22) are shown on the left-hand side with male parents (P1-P8) at the top. Percentages in cells indicate the number of individual F_1_ seedlings remaining after being exposed to Psa (*Pseudomonas syringae* pv. *actinidiae*) for four years in the field. The numbers in brackets are the number of individuals remaining from the total number of individuals that were planted from the family. For example, 4%, or two of 56 plants survived after four years in the field from the cross between P1 and P10.

**Figure 2:**
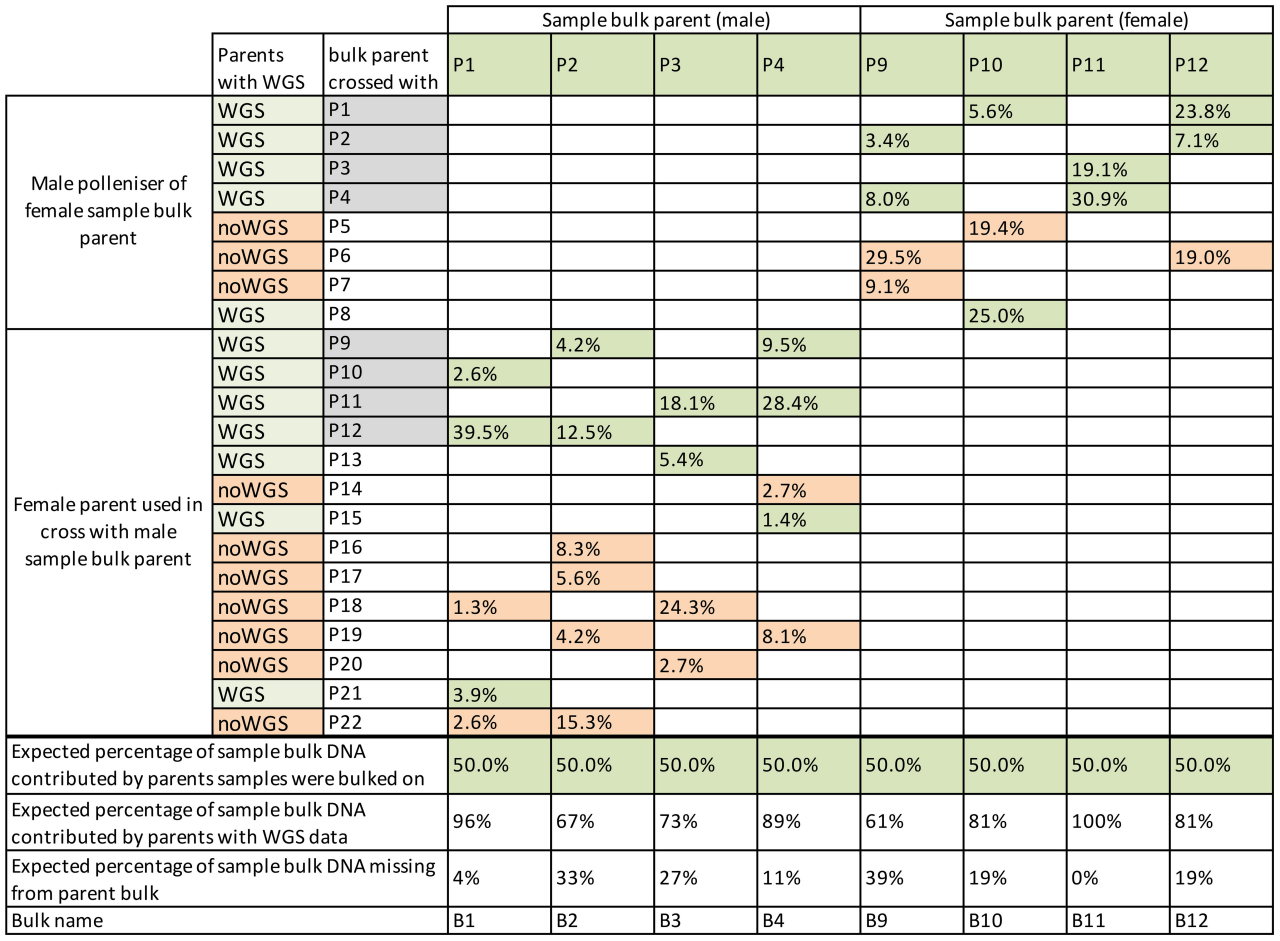
Percentage of each parent’s theoretical contribution to sample bulks. Bulks were based on the parents in columns, with parents contributing to the sample bulk in rows. Families from parents with grey-filled parent names were represented twice where the family was used in bulks based on male and female sample bulk parents. Parents with whole-genome sequence (WGS) data have cells filled in green, and those without WGS are filled in salmon. Parent bulks contained DNA only from the parents with green shading. The total theoretical DNA contribution missing from parents without WGS data in parent bulks is shown in the bottom row.

A diverse population of diploid *A. chinensis* var. *chinensis*, named “12x18”, was identified at Plant & Food Research, Kerikeri, New Zealand as a suitable population to meet the objectives of both aspects of this study. The parental seedling vines for this population were initially planted in 2015 and cultivated using a T-bar system, with a spacing of 0.75 m between each plant and 3 m between rows. The population was strategically distributed across three blocks, each spanning 4000 m^2^, with 6-m high hedging shelter belts serving as dividers and boundaries around the blocks. This population was naturally exposed to Psa, which was present in the Kerikeri orchard at the time of seedling planting. The exposure to Psa led to the development of symptoms in certain individuals including tip dieback, cane death, oozing from infected cankers, and in highly susceptible cases, complete plant death. To manage Psa symptoms, canes were removed if tip death or cane death was observed on a single cane. When more than three canes were infected with cankers or experienced cane dieback the entire vine was removed from the orchard. The structure of the 12x18 population was established by the crossing a diverse set of 12 female and 18 male parents from *Actinidia* germplasm, employing an incomplete factorial design that resulted in 63 families. A variable number of seedlings (33, 48, or 56) from each family were planted in the field after their initial establishment in pots. Fifty-nine families had individuals remaining after 4 years (**Figure 1**, **Table 1**, **Figure 2**). Between 2015 and 2019, severe Psa infections led to the removal of individuals from various families with a range from 63% to 100%. Of the families that had surviving individuals, only 25 had sufficient a sufficient number of individuals to be included in the current study. However, it was necessary to pool families based on their parentage due to the relatively low numbers in individual families.

**Table 1:** The number of males and females contributing to bulks in part one of this work.

Bulk	Number of males in bulk	Number of females in bulk	Total individuals in bulk	Percentage of males in bulk	Percentage of females in bulk
1	2	17	19	10.5	89.5
2	4	15	19	21.1	78.9
3	6	13	19	31.6	68.4
4	9	11	20	45	55
5	10	10	20	50	50
6	12	8	20	60	40
7	15	5	20	75	25
8	16	4	20	80	20
9	18	2	20	90	10

The percentage of males within each of the nine bulks ranged from 10.5% to 90%.

### Sample collection, DNA extraction and sequencing

The field sampling, DNA extraction processes, and sequencing methods were the same for both parts of this work. Sampling of plant material was done by placing a single leaf from each plant, destined for a bulk, into a plastic bag labelled with the bulk’s name. Leaf samples were taken from the third leaf from the growing cane tip and kept cold in a chilly box with ice while sampling. After field collection, the bulks of leaves were stored in a -80°C freezer before processing. A 10-mm diameter leaf disc was collected from the lamina of each leaf while frozen. All leaf discs from a bulk were finely ground together in liquid nitrogen with a pestle and mortar. DNA was extracted from the ground material with a Qiagen DNeasy^®^ Plant Maxi kit. To remove pectin from samples, DNA was precipitated by adding 1/10 volume sodium acetate (3 M, pH 5.2) to two times the volume (calculated after addition of sodium acetate) of at least 95% ethanol. Samples were incubated on ice overnight, then centrifuged at 14000 g for 30 min at 4° C. Supernatant was removed and rinsed with 70% ethanol, then centrifuged at 14000 g for 15 min. The supernatant was discarded, and the pellet dissolved in TE buffer (pH 8.0). TE buffer was made by adding 100 mL of 1M Tris-Cl (pH 8.0) to 20 uL of 0.5 M EDTA (pH 8.0) to 9.880 mL of reverse-osmosis water. DNA quality and quantity were checked using a Qubit^®^ 2.0. In samples with a low DNA quantity, extraction was repeated. In samples with low-quality DNA, identified by a 260/280 value of under 1.6, DNA was cleaned of pectin using a second ethanol precipitation step. In this step, DNA was precipitated in 98% ethanol and the DNA pellet was lightly massaged with a spatula against an Eppendorf tube wall to remove pectin within the DNA precipitate. A minimum of 1400 ng of DNA from each bulk was sent to the Australian Genome Research Facility (AGRF) for PCR free library preparation and whole-genome sequencing at 30x coverage with 150 bp paired-end reads using the Illumina NovaSeq 6000 platform.

Using this method of bulking leaf samples forgoes the step of extracting DNA from individual plants, quantifying the DNA from each extraction and adjusting the amounts so that each bulk contains an even amount from each contributing individual. However, it also introduces the risk of having a variable quantity of DNA added from each individual to a bulk and may therefore increase the error associated with analysing allele depth.

### Comparing the genomic difference between the parents contributing to bulks

Because the methods used in part two of this work could be influenced by the similarity of parents, the genomic distance between parents needed to be tested. The genomic distance between individuals can be analysed with principal component analysis by transforming genomic data into a Boolean vector, as described in Konishi et al. (2019). The variants from parents were used to identify the genomic distance between each parent with whole genome sequence (WGS) data.

### Bulking samples for part one of this study

The sensitivity of the sampling and bulk segregant analysis methods to detect a shift in allele frequency was investigated. This was achieved by using male and female F_1_ individuals from a mix of families from the 12x18 population that had parent P8 as the father (**Figure 1**, **Figure 2**). Female parents of these families included P10, P14, P15, and P17. The shift in allele frequency at the sex locus on chromosome 25 was tested between the nine bulks of DNA containing about 10%, 20%, 25%, 40%, 50%, 55%, 68.4%, 78.9% or 89.5% male contribution to the bulk from 19–20 individuals (**Table 1**).

### Bulking samples for part two of this study

Part two of this work investigated WGS data from bulks to detect whether there was a shift in allele frequencies within bulks of individuals that remained in families after exposure to Psa. This work was complicated because severe Psa infection had led to the removal of many individuals from all the families in the population. Because some families had very few individuals remaining, each of the eight sample bulks included resistant individuals from up to six families. These were bulked based on a single parent that contributed to all the families in the bulk (**Figure 1**, **Figure 2**). For example, the bulk B1 contained F_1_ families from crosses P1 x P10, P1 x P12, P1 x P18, P1 x P21, and P1 x P22.

Part two of this work differed from a typical BSA because there was no DNA from individuals that were removed because of Psa. Instead, the frequency of alleles in surviving individuals was compared against a bioinformatically generated bulk of data from parents contributing to the sample bulk. The bioinformatically generated parent bulks were used in place of bulks of individuals susceptible to Psa. Bulks like this can be used because the alleles in the parental bulks were representative of the families included in the bulks without selection. This methodology is similar to that done for selection mapping (Wisser et al., 2008; Matsumoto et al., 2017), but it has the drawback of assuming no other influences on allele transmission. The bioinformatically generated parent bulks were made by merging parental BAM files before variant call files (VCF) were made. However, not all parents that contributed to the families used in parental bulks had WGS data available (**Figure 2**, **Table 2**). As a result, the bulks of parents that contained some parents without WGS data would give a less accurate representation of the population before selection. The loss of information was particularly apparent in the bulk of B9, which had 29.5% of its theoretical DNA contribution missing from its P6 parent and 9.1% missing from the P7 parent (**Figure 2**). The missing data from parents would have resulted in some alternate alleles present in these parents not being included in the analysis. Unfortunately, once the pools were established during field sampling, the families with missing WGS data could not be removed from pools.

**Table 2:** Depth filter settings applied to data before Gprime analysis.

Sample bulk	Refence allele frequency	Minimum total depth	Maximum total depth	Depth difference	Minimum sample depth	Minimum genomic quality
B1	0.05	40	85	50	10	100
B2	0.05	35	85	50	10	100
B3	0.05	20	85	50	10	100
B4	0.05	40	150	50	10	100
B9	0.05	40	130	50	10	100
B10	0.05	40	90	50	10	100
B11	0.05	60	170	50	10	100
B12	0.05	30	100	50	10	100

Sample bulks B1-B12 retained the same reference allele frequency, allele depth difference, minimum sample depth and minimum genomic quality, but varied in the minimum total allele depth and maximum total allele depth depending on the distribution of depth data in each sample bulk.

### Bulk segregant analysis part one

To test the limits of the methods used to bulk samples and extract DNA to determine the architecture for Psa tolerance, WGS data for part one of this work were analysed with the QTLseqR package v0.7.5.2 (Mansfeld and Grumet, 2018). The analysis included nine bulks, with a varying number of males added to each bulk, were each compared with each other for 36 separate analyses, described further below. These comparisons were expected to present a QTL peak in the bulk segregant analysis at 1.6 Mb on chromosome 25. QTLs were expected on chromosome 25 because it contains the heterozygous dominant sex-determining *Shy Girl* gene that suppresses the feminisation of flower production to generate male flowers and thus a male plant (Akagi et al., 2018). But detection of QTL at the *Shy Girl* gene locus could only occur if the methods used were tolerant enough of the sampling and bulking methods, the effect of Psa on the families, and the relationship between the samples for the bulks, since these would have an influence on frequency of alleles between bulks. For example, if bulks with a 5% difference in male number were compared and QTL were consistently detected in comparisons with different backgrounds, and with a similar difference in male percentage between pools, it could be assumed that the methodology added 5% of error to the analysis.

Bulks of males were compared with each other because WGS data were unavailable for two of the five male bulk parents, P14 and P17. Thus, a bulk of these parents would not accurately represent the bulks of parental data. Instead, data from each of the nine bulks with a known percentage of males were compared with each other, resulting in 36 separate analyses that compared pairs of bulks. The difference between the percentages of males between bulk pairs varied between 5 and 79.5%.

Binary alignment files (BAM files) of WGS data were generated from compressed FASTQ formatted sequence files containing single read sequence output by aligning reads to an unpublished in-house reference genome of parent P8 by Roy Storey using BWA-MEM (Yao et al., 2020). The author completed the subsequent bioinformatics analyses using the R coding language. BAM files from separate flow cell lanes were merged with Picard “MergeSamFiles”. Samtools was used for sorting and indexing BAM files. Variant call files were generated using BCFtools mpileup with options including setting a minimum base quality of 20 and disabling probabilistic realignment to help reduce false SNPs caused by misalignments. Indel calls were excluded. Optional tags included the depth at each site, the depth of each allele, and the Phred-scaled strand bias P-value. Uncompressed output was piped to BCFtools call, which included the genomic quality and genotype posterior probability format fields and the multiallelic caller option. The resulting variant call files were indexed using BCFtools index. BCFtools query was used to split data into separate comma-separated value text files for each chromosome and exclude sites with a depth of less than 20 or greater than 200, and data were read into R/datatable.

The SNP index for each bulk pair to be analysed was calculated by dividing the alternate allele depth by the total read depth. The reference allele frequency was calculated by summing the reference allele depth of bulks being compared and dividing the result by the sum of the total depth of the bulks being compared. The delta-SNP index was calculated by subtracting the SNP index of the sample bulk from the parental bulk. The modified G statistic was calculated for each SNP based on the observed and expected allele depths (Magwene et al., 2011) and smoothed using a tricube smoothing kernel (Watson, 1964) in QTLseqR (Mansfeld and Grumet, 2018). The Gprime value was calculated from the tricube smoothed G statistic by taking the average weight of the physical distance across the neighbouring SNPs within the 1-Mb window. This approach accounted for linkage disequilibrium and minimized the noise attributed to SNP calling errors (Magwene et al., 2011). SNPs were filtered using the QTLseqR package selecting a reference allele frequency of 0.05, a minimum total depth of 60, a maximum total depth of 160, an allele depth difference of less than 50 between bulks, a minimum sample depth of 10 and a minimum genomic quality of 100. The QTLseqR analysis package had the bulk size set to 20 individuals with the Gprime window size set at 1 Mb. Because the adjusted p statistic threshold failed to detect peaks with a low difference in allele frequency, the genomic position of the top 0.5% of SNPs were used as the peak locus.

### Bulk segregant analysis part two

Part two of this work used the same bioinformatics pipeline as part one of this work to generate VCF files of sample bulks. However, part two of this work differed from part one because a modified BSA approach was used for bulk creation and sample bulks were compared against the bioinformatically generated bulk of WGS data from parents that contributed to the bulked families (**Table 1**). Bulks were selected for analyses based on the presence of WGS data for parents and having greater than ten individuals available for sampling. These parent bulks were created by merging BAM files from parents using samtools-merge. VCF files were created with BCFtools-mpileup using the same options as in part one of this work.

Before the sample bulks and parent bulks could be compared, calculations based on SNP data from VCF files were performed. VCF files were read into R/datatable, and a SNP index was calculated for each bulk by dividing the alternate allele depth by the total read depth. The reference allele frequency was calculated by summing the reference allele depth of the sample bulk and the parent bulk, and dividing the result by the sum of the total depth of the bulks being compared (Mansfeld and Grumet, 2018). The delta-SNP index was calculated by subtracting the SNP index of the sample bulk from the parental bulk (Mansfeld and Grumet, 2018).

The data preparation for analysis in QTLseqR was done similarly to that done in part one. First, BCFtools-query split data into separate.csv files for each chromosome and excluded sites with a depth of less than 20 or greater than 200. The SNP index per bulk was calculated by dividing the alternate allele depth by the total read depth. Unlike in part one, in part two the reference allele frequency was calculated using the sum of reference allele depths of sample bulks and the result was divided by the sum of the total depth of the parental bulks. The delta-SNP index was calculated by subtracting the SNP index of the sample bulk from the parental bulk.

**Figure 3:**
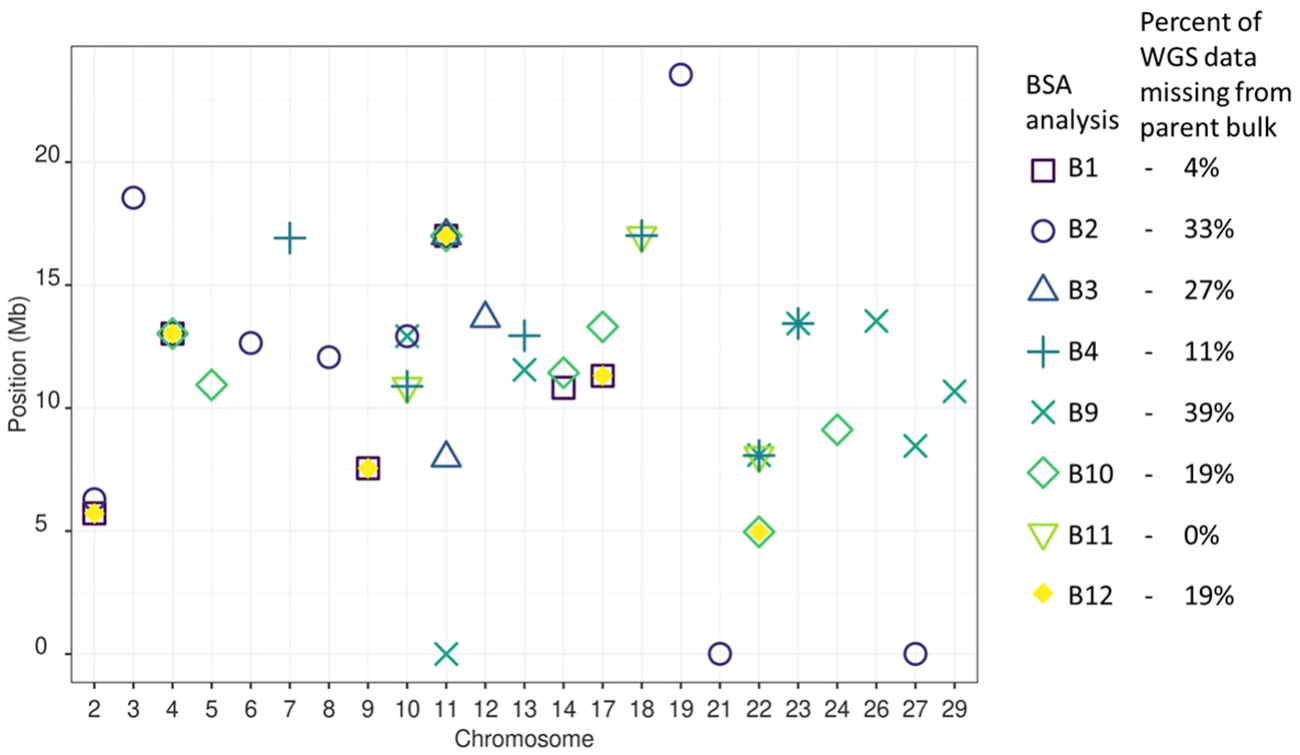
BSA QTL peak positions for Psa resistance between all analyses. Thirty QTLs were detected among the eight bulks analysed. The BSA presenting the most unique QTL were B2, B9, and B10. The BSA presenting no unique QTL were B11 and B12. A single QTL site was common between four BSA on Chromosome 11 at 16.95 Mb from B1, B3, B10 and B12. Four QTL sites were common between three BSA on chromosome 2 at 5.35 Mb, chromosome 4 at 13.02 Mb, Chromosome 17 at 11.31 Mb, and Chromosome 22 at 8.07 Mb. Six QTL sites were found in common between two BSA on Chromosome 9 at 7.55 Mb, Chromosome 10 at 10.89 and 12.92 Mb, Chromosome 14 at 11.13 Mb, Chromosome 17 at 11.31 Mb, Chromosome 18 at 17.01 Mb, Chromosome 22 at 4.97 Mb, and Chromosome 23 at 13.44 Mb. The 17 remaining QTL sites were found in a single BSA.

Data from each of the eight sample bulks were compared with their parent bulk using the Gprime analysis portion of the QTLseqR package (Mansfeld and Grumet, 2018). Gprime analysis was used because the average G values across SNPs in the 1-Mb sliding window reveal the signal of divergence in allele frequency between bulks that are conserved between closely linked sites (Magwene et al., 2011; Mansfeld and Grumet, 2018). Using the G value reduces the influence of random noise due to variable sequencing read coverage (Mansfeld and Grumet, 2018). Within the QTLseqR package, SNPs were filtered by depth for each comparison depending on the data distribution. Minimum and maximum total depth were set to remove SNPs of extremely low and extremely high frequency (**Table 2**) (Mansfeld and Grumet, 2018). Filtering SNPs by read depth helps remove SNPs with low confidence due to low coverage, or remove SNPs in repetitive regions that would have an artificially inflated read depth (Mansfeld and Grumet, 2018). Settings for the Gprime analysis method were as follows: the sliding window size was set at 1 Mb, the outlier filter was set as “deltaSNP”, and the filter threshold was set at 0.4. The resulting Gprime values for each SNP site were plotted with the top 0.5% of Gprime values and peak loci for each bulk plotted in **Figure 3**.

## Results

**Figure 4:**
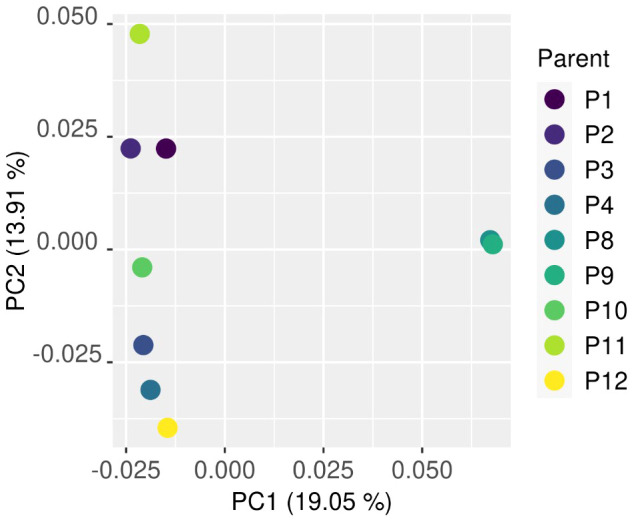
The genomic distance between parents that contributed to bulks analysed by principal component analysis. A close relationship among the half-siblings P8 and P9 was found with a greater distance between P8 and P9 and the other individuals at PC1. The close relationship between P8 and P9 remained at PC2, with a close relationship between P1 and P2.

The PCA comparing the parents that contributed to bulks that also had WGS data showed a close relationship among the half-sibling individuals P8 and P9, with a greater distance between P8 and P9 and the other individuals at PC1. PC2 showed an even distribution of genomic relationship between the remaining individuals with the exception of P1 and P2, which had minimal genomic distance on PC2 (**Figure 4**).

**Figure 5:**
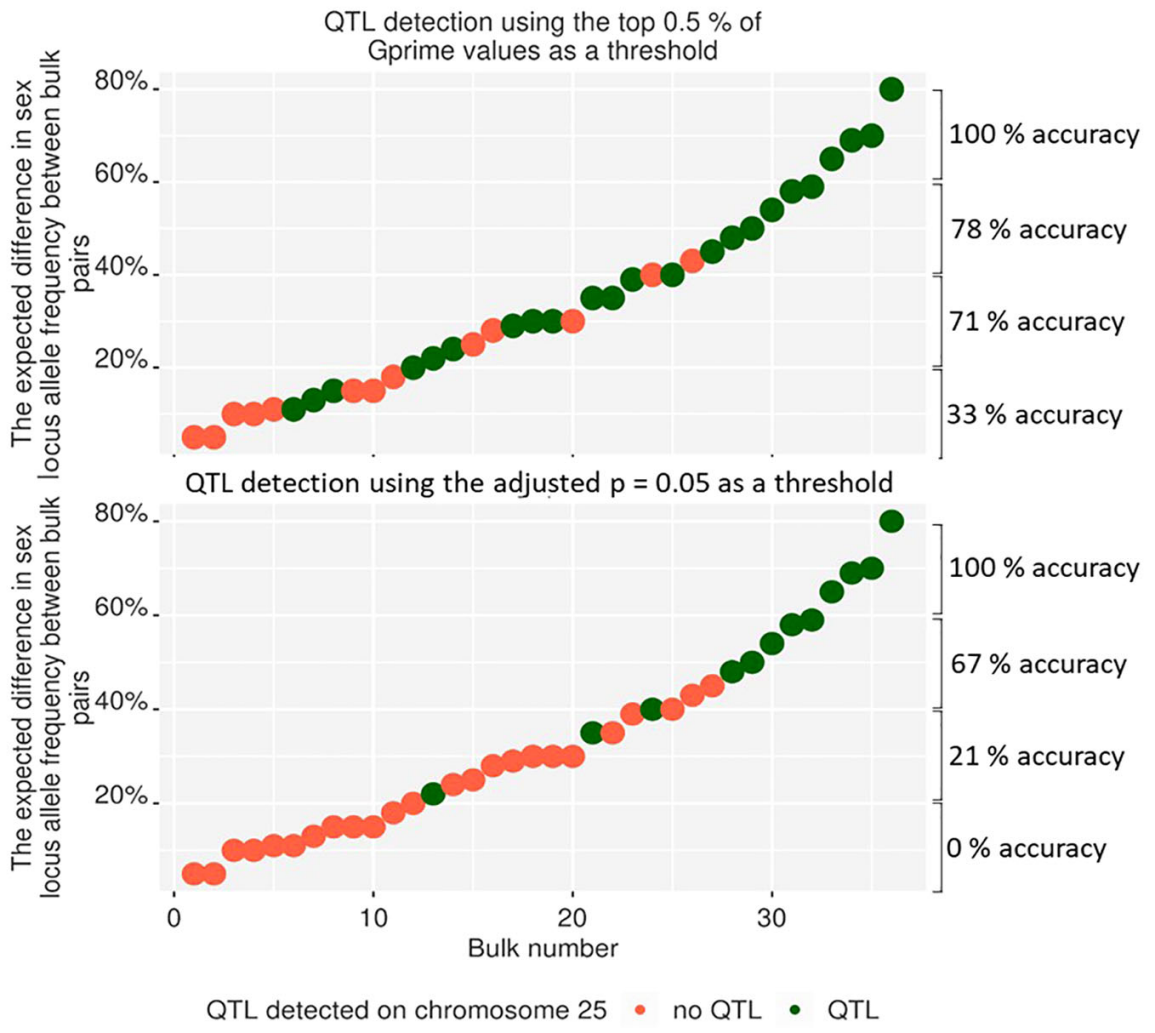
The detection of QTLs on chromosome 25 from pairwise comparisons between nine bulks with a different percentage of males added to each bulk. This figure presents only QTL from chromosome 25, but the analysis was completed over the whole genome. Using the top 0.5% of SNPs over the whole genome (top) to detect QTL peaks gave fewer false-negative results than QTL peaks, which were deemed statistically significant using an adjusted p-value of greater than 0.05. When using the top 0.5% threshold, the detection accuracy was estimated to be 33%, 71%, 78%, and 100% for an expected difference in allele frequency between bulks of 0–20%, 20–40%, 40–60%, and 60–80%, respectively. When using the adjusted p=0.05 threshold, the detection accuracy was estimated to be 0%, 21%, 67%, and 100% for an expected difference in allele frequency between bulks of 0–20%, 20–40%, 40–60%, and 60–80%, respectively.

The methods used for the BSA in this work differed from the standard methods used for BSA in QTLseqR (Mansfeld and Grumet, 2018). Therefore, the sensitivity of these methods to detect QTL in bulks of *A. chinensis* var. *chinensis* needed to be tested. Part one of this work tested the resolving power of the methods by making 36 pairwise comparisons at the sex loci on chromosome 25 among the nine bulks of individuals with a differing percentage of males. However, QTLs were detected in only 12 of the 36 bulk comparisons when using the adjusted p = 0.05 threshold (**Figure 5**). Increasing the threshold to adjusted p = 0.1 included more QTL peaks, but also significantly increased the signal-to-noise ratio. Because the adjusted p-value based threshold could be caused by the alternative method of bulking multiple families or lack of inclusion of some parents in the bioinformatically generated bulk of parents, the significance threshold was changed to use the top 0.5% of Gprime values. Using the top 0.5% of Gprime values allowed QTL detection from BSA with greater accuracy in bulks, detecting QTL at the sex-linked gene locus in 19 of the 36 bulks analysed. However, using the top 0.5% of Gprime values as a threshold of significance for QTL detection also has the disadvantage of missing smaller peaks for QTL in BSA plots where the signal for selection for some QTL was strong and covered a large range of loci. For example, the P3 bulk may have signal for selection on chromosomes 25 and 29, but the peaks on chromosomes 11 and 12 hold the top 0.5% of Gprime values.

To determine the effect of Psa on *A. chinensis* var. *chinensis* alleles in an incomplete factorial population, in part two of this work, samples that survived Psa were assigned to bulks based on families with a parent in common (**Figure 1**, **Figure 2**). Using BSA, the eight sample bulks were compared against bioinformatically created bulks of parental WGS data. The eight resulting BSA, presented in **Supplementary Material**, identified sites of higher frequency in the sample bulk compared to the parent bulk, potentially caused by selection for resistance to Psa. The QTL presented as higher Gprime values in the resulting BSA plots, with the top 0.5% of Gprime values considered significant QTL. These significant QTL were summarised between bulks in **Figure 3**.

In theory, the variants for resistance to Psa had a maximum potential selection of 50%. For example, in the case of a cross ab x cc with resistance associated with the ‘a’ variant, if there was strong selection for the ‘a’ variant in all seedlings, the resulting family containing ac variants would have a variant frequency 50% higher than if there was no selection producing ac and bc variants. The b variant will also decrease in frequency by 50%.

## Discussion

Psa is one of the most destructive diseases affecting kiwifruit, with a broad range of susceptible and tolerant *A. chinensis* var. *chinensis* genotypes. However, QTLs for Psa resistance have been published within only two families to date (Tahir et al., 2019; Tahir et al., 2020), potentially leaving many alleles for resistance to Psa undiscovered. Typically, QTL mapping methods would be used to investigate loci for a polygenic trait such as Psa (Jansen, 1996; Lefebvre and Palloix, 1996). However, accurately identifying traits influenced by more than one locus with QTL mapping is a costly and resource-intensive process requiring large replicated families specially developed for this purpose (Wisser et al., 2008; Soto-Cerda and Cloutier, 2012; Tahir et al., 2018; Gupta et al., 2019; Tahir et al., 2019). The work presented here overcame the limitations of the typical QTL mapping process by using bulks of diverse F_1_ families in a modified BSA. This approach further increased the utility and cost-effectiveness of the typical BSA methods by analysing multiple small families in a single bulk, decreasing the sampling complexity, reducing the DNA extraction time and cost, reducing sequencing costs, and increasing the breadth of Psa resistance alleles that could be detected within a bulk.

Bulked DNA has been analysed using BSA methods for a wide range of species and traits (Michelmore et al., 1991; Song et al., 2017; Dong et al., 2018; Sun et al., 2018; Xin et al., 2020; Li and Xu, 2022; Shen and Messer, 2022). The approach taken here used BSA analysis methods to identify alleles for Psa resistance by measuring the shift in allele frequency of an *A. chinensis* var. *chinensis* population that had many individuals removed from established families due to Psa. The selective sweep of susceptible individuals provided a prime opportunity to measure the effect of this selection on the alleles that remained within those *A. chinensis* var. *chinensis* families. However, because susceptible plants were not sampled prior to loss, DNA was not captured from susceptible individuals, which a typical BSA would use as the comparison bulk (Michelmore et al., 1991; Li and Xu, 2022). The lack of a susceptible bulk was compensated for in this modified BSA by using a bulk of parent WGS data as a population not exposed to selection pressure for Psa resistance. To our knowledge this is the first time a bulk of parents has been used as a substitute for a bulk in a BSA analysis and the first time QTL for kiwifruit resistance to Psa have been identified within a diverse range of families.

### Testing the sensitivity of the modified methods

Part one of this work tested the ability of the modified BSA method to analyse the effects of Psa in kiwifruit by identifying a known locus through detecting a shift in allele frequency between bulks. This test was required because the families were sampled and extracted as bulks in a unique way (**Figure 1**). The modified method involved sampling leaves for a bulk from the field into a single bag and extracting DNA from all leaf samples within the bulk by first grinding them all together before DNA extraction. This significantly increased the speed of sampling, sample tracking and DNA extraction, but variance among leaves could still occur due to differences in the number and size of cells, ratio of mitotic to interphase nuclei, or differing structures or biochemical composition of the plant cells (Marsal et al., 2013). As a result, changes in allele frequency could have been created between samples due to a variable amount of DNA extracted from each leaf sample. Testing the effect of the sampling methods and their integration with the analysis methods on allele detection found that the detection of an allele frequency shift of over 10% was effective for detecting alleles under strong selection for Psa resistance for part two of this work. The detection accuracy was enhanced by modifying the threshold of detection for QTL. The modified thresholds allowed the detection of a 20–40% shift in male allele frequency with an accuracy above 71% (**Figure 5**), and a shift in allele frequency above 60% could be detected with 100% accuracy. This confirmed that the modified technique could detect large changes in allele frequency. Because strongly selected alleles can cause a shift in allele frequency of up to 50% within a family (Miko, 2008), this increased the confidence that sites of strong selection could be detected in part two of this work. However, these methods may not detect small shifts in allele frequency.

### QTL for resistance to Psa

**Figure 6:**
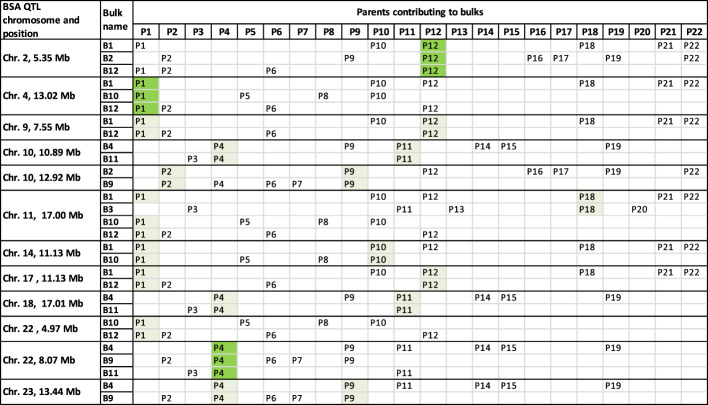
Chromosomes and sites with more than one QTL for resistance to Psa in common between analyses. The parents contributing alleles to each QTL can be determined for some peaks by analysing the families that contribute to each bulk. The QTL, highlighted in green, from Chromosome 4 was likely from the parent P1. But the indication of parent P1 being the main contributor to QTL peaks on Chromosome 14 at 11.13 Mb and Chromosome 22 at 4.97 Mb may be misleading as the bulk B10 had a low contribution from parent P1. This may not have had a strong enough signal to present as a peak unless the loci were shared with another parent such as P12. The QTL on Chromosome 2 at 5.35 Mb was likely from parent P12, and the peak on Chromosome 22 at 8.07 Mb was likely from parent P4. Other parents contributing to QTLs in light green had two individuals that could have contributed to the QTL. Chromosome 11 had no parental contributors to bulks in common with the QTL at that position, despite four bulks presenting QTL at that site. It is possible that the parents, P1 and P18, contained the same alleles for Psa resistance on Chromosome 11 at 16.95 Mb.

A typical BSA groups together loci that differ in allele frequency between sample bulks segregating for a trait of interest (Michelmore et al., 1991; Li and Xu, 2022). However, part two of the approach taken here modified the bulking strategy of a typical BSA. Each sample bulk was made up of multiple diverse families that had DNA extracted from all individuals in the bulk simultaneously as a single sample. Part two of this work also differed from a typical BSA because no individuals culled due to Psa infections were sampled to make a comparison bulk. Instead, a bulk of parental alleles was bioinformatically generated using WGS data from parents of the families under investigation. The alleles with a higher frequency in the sample bulk compared to the parent bulk were assessed against the significance thresholds established in the preliminary trial to identify alleles for Psa resistance. Between the eight sample bulks that each contained three to seven families, 30 QTLs for resistance to Psa were identified. Twelve of the 30 QTLs were detected in more than one bulk, with one locus on Chromosome 11 at 16.95 Mb detected among four bulks (**Figure 6**).

Alleles for Psa resistance have previously been published in a large diploid *A. chinensis* var. *chinensis* family (Tahir et al., 2019). That study identified two QTL using field scores for Psa resistance. One of the loci identified was in an identical location to the locus found in this work on Chromosome 22 at 4.967790 Mb from two bulks, B10 and B12. Because the parent P8 was used in the bi-parental population by Tahir et al. (2019), and bulk B10 from this work also contained the parent P8, it seems likely that this locus for resistance to Psa is coming from parent P8. The other locus for Psa resistance detected by Tahir et al. (2019) using field scores, found on Chromosome 27 at 4.305319 Mb, was not detected in this study. The lack of detection of this locus may have been because the cultivar ‘Hort16A’ that identified as the parent that contributed Psa resistance to the bi-parental family was not included in this study. A parent of ‘Hort16A’, included in this study, named parent P13, was included in bulk B3 but the contribution of P13 to this bulk was low at 5.4%. It would be expected to have a peak if the resistance allele was strongly selected for in the B3 bulk, but it is more likely that the other parent of ‘Hort16A’, named CK15_01 by Tahir et al. (2019), was the contributor of the resistance QTL found by Tahir et al. (2019) on Chromosome 27 at 4.305319 Mb.

The commonality of resistance allele sites among some of the different BSA bulks may reflect the inclusion of common parents that contributed to those bulks. This commonality of QTL sites can give insight into which of the parents were likely to be contributing the alleles under selection at some of the QTL. Looking at the peaks that are at the same site on the same chromosomes in different bulks (**Figure 6**) allows us to infer the most likely parents that were contributing the alleles to those QTL. For example, the QTL on Chromosome 2, at 5.35 Mb, is shared between bulks B1, B2, and B12, indicating that parent P12 is likely to be the source of alleles in higher frequency in those bulks (**Figure 6**). Similarly, the parent P4 is likely the source of the QTL on Chromosome 22 at 8.07 Mb and the parent P1 is likely the source of the QTL on Chromosome 4 at 13.02 Mb. The parent P1 also appears to be the contributing parent to the QTL on Chromosome 22 at 4.97 Mb. However, this prediction of the P1 parent contribution to QTL at 4.97 Mb on Chromosome 22 may be inaccurate as the P1 parent makes up only a small proportion of the bulk B10 (5.6%). Instead, both the parents P6 and P8 may have contributed this QTL to these bulks. Parent P8 was used as one of the parents in a biparental mapping family for Psa resistance in a study by Tahir et al. (2019) and parents P6 and P8 are related. Tahir et al.’s study (2019) also identified the same QTL at 4.97 Mb on Chromosome 22 derived from parent P8. Eight other QTLs could have their parent contributors narrowed down to only two parents since both were shared between bulks and sites (**Figure 6**).

**Figure 7:**
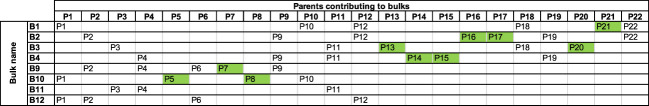
Bulks with parent contributors unique to each bulk. Parents that were represented in a single pool are highlighted in green.

In cases where a QTL was detected in only one bulk, the allele responsible may have been contributed by a parent unique to that bulk (**Figure 7**).

Therefore, the QTL peaks on Chromosomes 11 at 0.36 Kb, 13 at 11.55 Mb, 26 at 9.12 Mb, 27 at 8.46 Mb and 29 at 10.68 Mb from bulk B9 were likely contributed by the parent P7. The remaining QTL sites from bulks B2, B3, B4 and B10 each had two unique parents that likely contributed to the detected QTL: namely, parents P16 or P17 contributed to Chromosomes 3 at 18.56 Mb, 6 at 12.65 Mb, 8 at 12.07 Mb, 19 at 23.56 Mb, 21 at 7.45 Kb, and 27 at 4.76 Kb, parents P13 or P17 to Chromosomes 11 at 7.97 Mb and 12 at 13.65 Mb, parents P13 or P20 to Chromosomes 7 at 16.92 Mb and 13 at 13.65 Mb from bulk B3, and parents P5 or P8 likely contributed to resistance alleles on Chromosomes 5 at 10.95 Mb, 17 at 13.31 Mb and 24 at 9.12 Mb from bulk B10. It was reassuring that the B11 and B12 bulks, which had no parents unique to the bulk, had no unique resistance allele sites attributed to them.

Further information about parent contributors to resistance alleles can be gained by identifying the parents in common among bulks that contributed to these alleles. This approach identified the likely parental contributors to three resistance alleles on Chromosomes 2, 4, and 22 from parents P1, P4 and P12, respectively (**Table 3**).

**Table 3:** The commonality of parents contributing resistance loci among bulks.

Parent contributing to bulks	P1	P4	P12
**Bulks containing parents**	B1	B4	B1
	B10	B9	B2
	B12	B11	B12
**QTL position**	Chr. 4, 13.02 Mb	Chr. 22, 8.07 Mb	Chr. 2, 5.35 Mb

The parents that contributed specific alleles can be inferred where multiple bulks have resistance alleles at the same site that shared parents among those bulks. Parent P1 was the likely contributor to the resistance allele on Chromosome 4. Parent P4 was the likely contributor to the resistance allele on Chromosome 22, and Parent 12 was the likely contributor to the resistance allele on Chromosome 2.

### Effects of the selective sweep for Psa tolerance alleles

Within each sample bulk, the families that had more individuals surviving Psa contributed more DNA to the bulk compared to those with fewer surviving individuals included in the same bulk. When performing the BSA, bulks with a skewed family representation may have preferentially identified loci from the families with more individuals in the bulk. This is likely because a higher amount of DNA contributed to a site from a particular parent increases the read depth of a locus unique to that parent compared to the bulk of parents. This is what was expected for the resistant alleles, but the families that had fewer surviving individuals would be under-represented in the bulk and therefore the Gprime value may be lower for these loci. The lower Gprime value may be excluded at a locus of interest due to families with greater representation presenting higher Gprime values over a greater number of loci.

The individuals that contributed to bulks all survived the selective sweep caused by Psa. The selective sweep would have exerted strong selection for alleles linked to resistance loci, such as those at 16.95 Mb along Chromosome 11 in bulks 1, 3, 10, and 12. Conversely, the selective sweep would have significantly reduced the frequency of alternative alleles at those loci (Nielsen et al., 2005). With strong selection pressure for an allele from a parent contributing to the family, the other allele would be effectively eliminated from the population at that locus. However, changes in allele frequency can also be indirectly caused through genetic correlations from linkage disequilibrium (Barrett and Hoekstra, 2011; Kemppainen et al., 2017) and genetic drift (Conolly et al., 2008). The Gprime method of BSA was implemented to adjust for the effects of linkage disequilibrium (Magwene et al., 2011), but genetic drift could have skewed the results, particularly in families with poor representation in the bulk. This is because the families with poor representation in the bulk are also a poor representation of that family, which will predispose the alleles from these families to genetic drift (Magwene et al., 2011). Similar effects will have occurred at genomic regions linked to the alleles for Psa resistance (Robertson, 1970). However, because the effects of genetic drift are assumed to be random throughout the genome (Conolly et al., 2008), the effect of genetic drift on the results from this work are assumed to be minor and resistance alleles detected in multiple bulks are unlikely to be caused by genetic drift.

Identifying the parents that contributed the Psa resistance loci to each bulk will help with family-based breeding strategies (Hinds et al., 2005). Although the families that contributed the largest number of individuals to bulks are likely to be those that are contributing the alleles for resistance in each bulk, these resistance alleles could be coming from one or many parents. An attempt was made to identify the parents contributing resistance to the sample bulks analysed, but a combination of the missing parental WGS data and the way the bulks were constructed limited the information available. This could be overcome by developing markers to target the loci with high Gprime values. The markers could then be used on DNA from parents to identify which parents contributed these resistance alleles and enable marker-assisted selection for Psa resistance in families related to these parents. Identifying parents that contributed causal resistance alleles to a bulk could also be done by identifying alleles of higher frequency from the BSA under the QTL that were private to a single parent (Hinds et al., 2005). However, this was not possible in this work because many of the parents did not have WGS data available (**Figure 2**). Identifying haplotypes for each parent would also be beneficial by allowing the haplotype sequence of each parent to be matched with loci with high Gprime values under QTL. This would be informative for the parents of bulks B1, B4 and B11, but WGS data from parents P5, P6, P7, P16, P18, P19 and P22 would still be needed to generate haplotype sequences to identify the parents contributing to QTL in bulks B2, B3, B9, B10, and B12.

### QTL detection accuracy of loci from parents that contributed a small or large percentage of DNA to bulks

Accurately detecting loci for resistance to Psa in part two of this work was dependent on the percentage of alleles that each family contributed to the bulk. Families that contributed more than 20% to a bulk and had strong allelic selection pressure on alleles unique to that family are likely to have those alleles present as a QTL in 72% of analyses (**Figure 5**). But, families that had strong selection pressure on alleles unique to that family and contributed 10–20% to a bulk were likely to present as a QTL in only 40% of analyses. Families that contributed less than 10% to a bulk were unlikely to present any QTL in the BSA, even with strong selection pressure on alleles unique to that family (**Figure 5**). Therefore, it is unlikely that families with poor representation in the sample bulk contributed to QTL in part two of this work. However, these families with low contribution to bulks may contribute the same resistance loci as other parents included in the bulk, adding to the significance of those sites. The lack of representation from the families contributing less than 10% to a bulk was due to the dilution created by other families that made up a bulk. For example, if an individual contributed 10% to a bulk and 50% of alleles were from one heterozygous parent under strong selection for Psa resistance, the resistance allele might be in all the individuals sampled from that family. Conversely, where resistance alleles are shared between families contributing to a bulk, their contribution to the sample bulk would stack, making their representation in the sample bulk 50% higher than in the parental bulk.

### Future research

Future projects could improve upon the methods used in part one of this work. Sampling of individuals for the bulks containing different sex ratios should have been done on families where parental WGS data were available for all of the parents contributing to the families used in each bulk. If this were done when sampling in part one this work, the sample bulks with a differing number of males and females in each bulk could have been compared against the bioinformatically created bulk of parents to match part two of this work. However, this was not done because some of the parents that contributed to these bulks did not have WGS data. Having an accurate test of the methods would alleviate the concern that the inferences made in part one of this experiment reflect only the allelic variance included in the sampling and DNA extraction methodology and may not accurately reflect the influence of the BSA methods on identifying alleles for Psa resistance in part two of this work. If part two of this work were repeated, collecting leaf material from plants before being culled would create a better match to a typical BSA (Li and Xu, 2022) and allow the creation of a bulk of alleles that were being selected against instead of using a pool of parental WGS data as the comparison bulk. Also, collecting leaf samples from all plants before they were affected by Psa would enable the creation of an unselected bulk, a negatively selected bulk, and a positively selected bulk. Comparing the positively selected bulk of individuals resistant to Psa against the unselected bulk may allow the identification of alleles associated with resistance to Psa. Comparing the negatively selected bulk of individuals that were removed because of Psa against the unselected bulk may allow the identification of alleles associated with susceptibility to Psa. Integrating the crossing and sampling plans would increase the accuracy of analyses because the unselected bulk would be a better representation of the alleles within the family than that of bulks of WGS data from parents.

The two parts of this work showed that finding multiple alleles for resistance to Psa can be achieved using BSA of bulks containing multiple families while greatly simplifying field sampling, DNA extraction, and reducing sequencing costs. To our knowledge, this is the first time DNA has been extracted as a bulk for a BSA instead of quantifying DNA from each individual separately and bulking the resulting DNA. This is also the first time BSA has been applied to bulks of families, where the comparison bulk was made up of a bioinformatically generated bulk of parental WGS data to identify loci affecting the target trait. Utilising the alleles for Psa resistance found in this work as selection criteria in breeding programmes may enable faster breeding of cultivars with greater resistance to Psa than without marker-assisted selection and provide an opportunity to stack resistance loci to create a more robust resistance to Psa in future cultivars.

## Data availability statement

The data presented in the study are deposited in the http://www.ncbi.nlm.nih.gov repository, accession number PRJNA1077747.

## Author contributions

CF: Conceptualization, Data curation, Formal Analysis, Methodology, Project administration, Writing – original draft, Writing – review & editing. VS: Supervision, Writing – review & editing. RS: Data curation, Writing – review & editing. MD: Writing – review & editing, Data curation, Visualization. PD: Supervision, Writing – review & editing.
