# Supplementary material for: Mapping QTL associated with resistance to Pseudomonas syringae pv. actinidiae in kiwifruit (Actinidia chinensis var. chinensis)
Source: Front Plant Sci. 2024 Mar 26;14:1255506. doi: 10.3389/fpls.2023.1255506 (PMC11003357; doi:10.3389/fpls.2023.1255506)
Supplement: Supplementary file 1 [file DataSheet_1.docx]

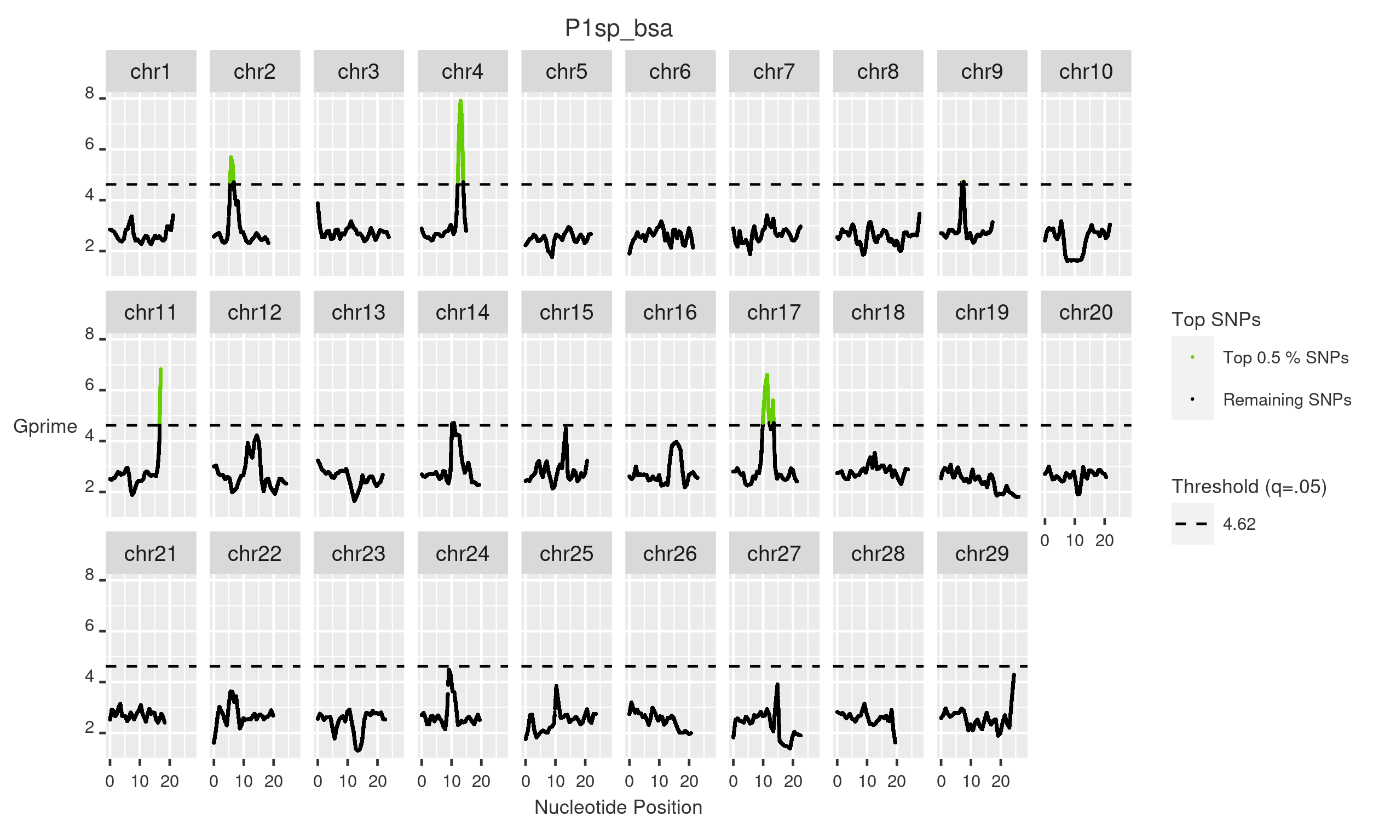


Bulked segregant analysis, B1, between sample bulk P1 and a bulk of parental WGS data contributing to P1.

Each analysed SNP site presents as a Gprime value analysed in a 1-Mb sliding window. Higher Gprime values reflect a higher frequency of P1 bulk alleles at certain genome positions than the bulk of parental WGS data. Green points represent the top 0.5% of Gprime values, with the remaining points coloured black. The threshold of the adjusted p = 0.05 is shown as a dashed line. QTL peaks for B1 were found on Chromosome 2 at 5.45 Mb, Chromosome 4 at 12.1 Mb, Chromosome 9 at 7.35 Mb, Chromosome 11 at 16.65 Mb, Chromosome 14 at 10.65, and Chromosome 17 at 10 Mb.


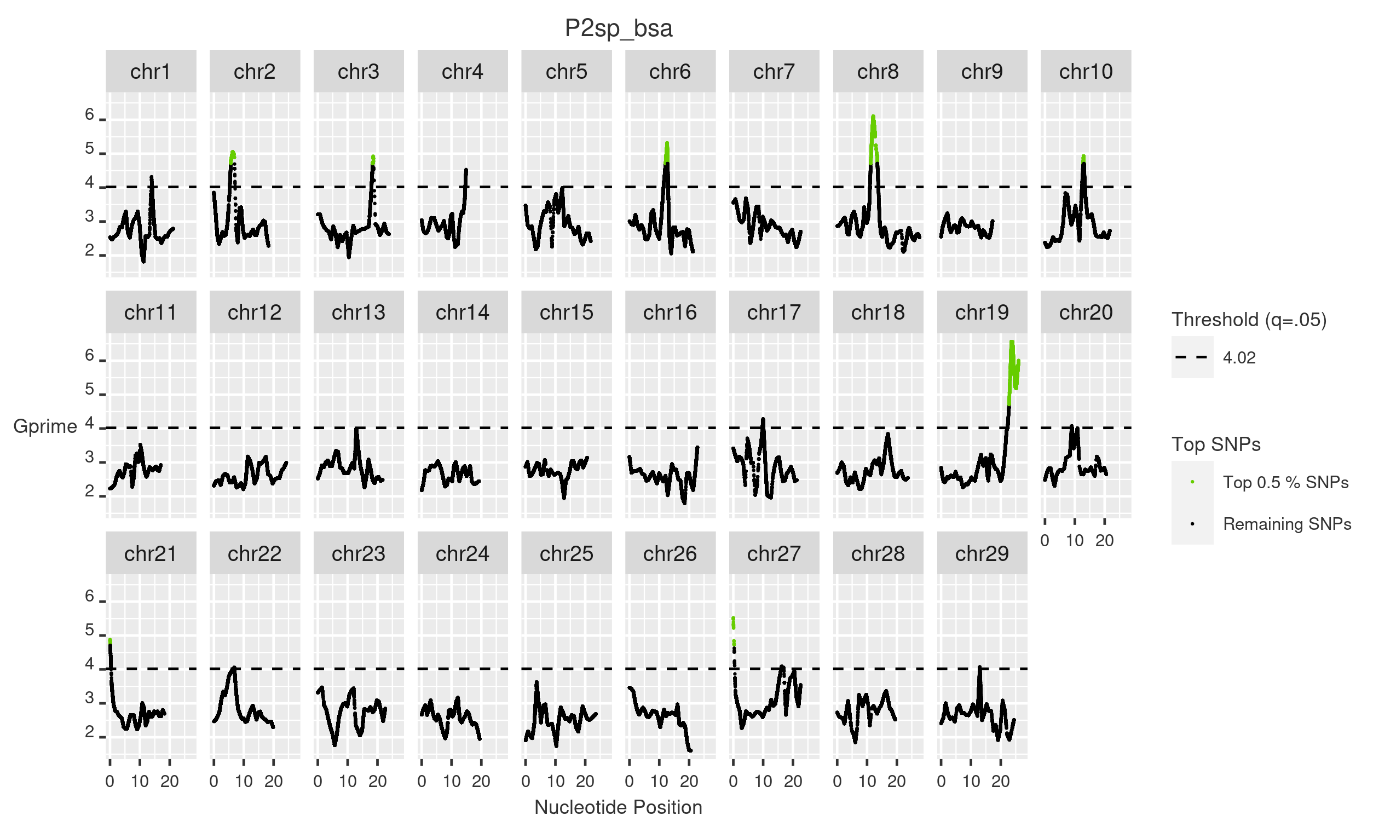


Bulked segregant analysis, B2, between sample bulk P2 and a bulk of parental WGS data contributing to P2.

Each analysed SNP site presents as a Gprime value analysed in a 1-Mb sliding window. Higher Gprime values reflect a higher frequency of P1 bulk alleles at certain genome positions than the bulk of parental WGS data. Green points represent the top 0.5% of Gprime values, with the remaining points coloured black. The threshold of adjusted p = 0.05 is shown as a dashed line. QTL peaks for B2 were found on Chromosome 2 at 5.67 Mb, Chromosome 3 at 18.42 Mb, Chromosome 6 at 12.12 Mb, Chromosome 8 at 11.25 Mb, Chromosome 21 at 0.01 Mb, and Chromosome 27 at 0.01 Mb. The threshold of significance was lower than the top 0.5% of SNPs.


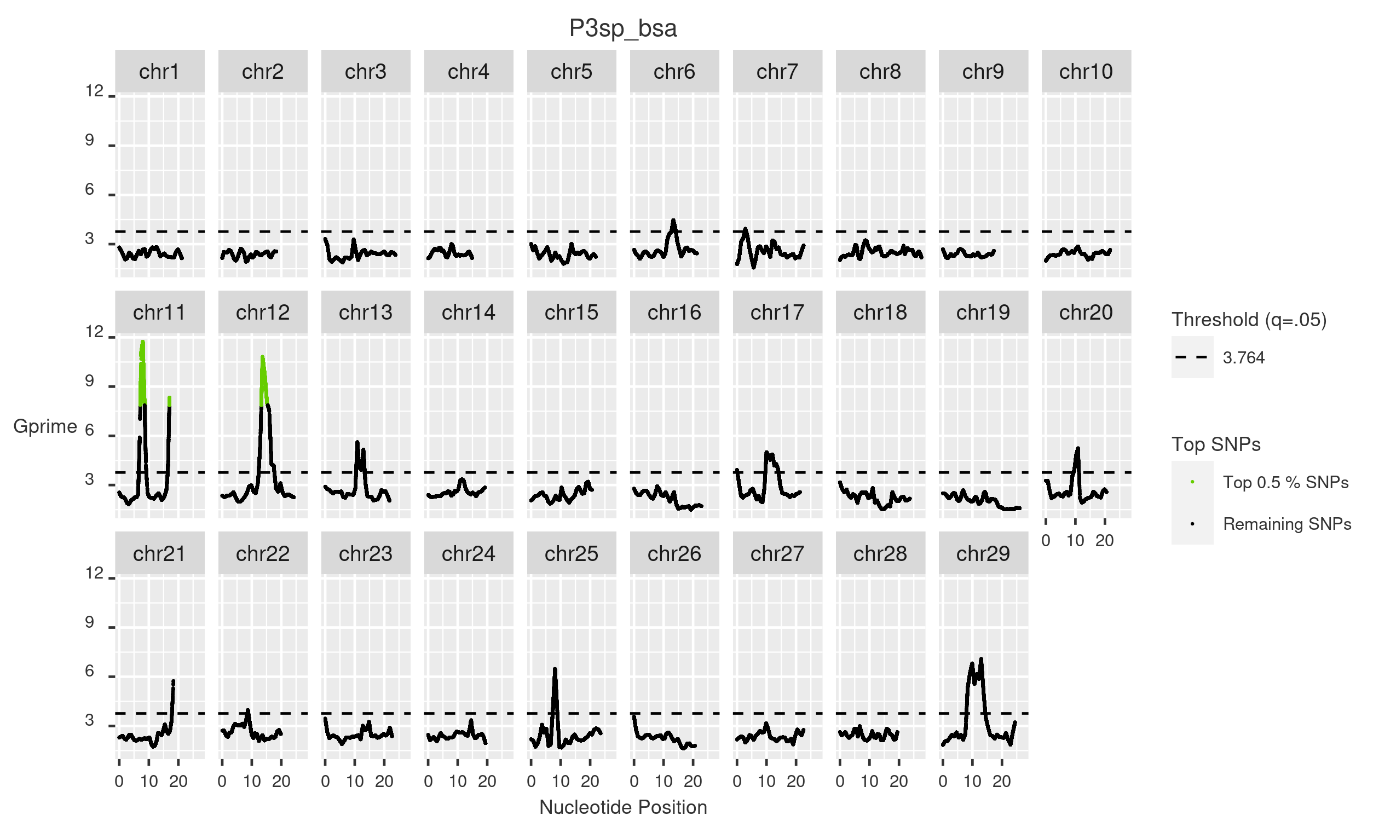


Bulked segregant analysis, B3, between sample bulk P3 and a bulk of parental WGS data contributing to P3.

Each analysed SNP site presents as a Gprime value analysed in a 1-Mb sliding window. Higher Gprime values reflect a higher frequency of P1 bulk alleles at certain genome positions than the bulk of parental WGS data. Green points represent the top 0.5% of Gprime values, with the remaining points coloured black. The threshold of adjusted p = 0.05 is shown as a dashed line. Three QTL peaks for B3 were found: two on Chromosome 11 peaking at 7.00 Mb and 16.95 Mb, and one at 13.22 Mb on Chromosome 12. The threshold of significance was lower than the top 0.5% of SNPs for this analysis, reflecting the strength of QTL on Chromosomes 11 and 12.


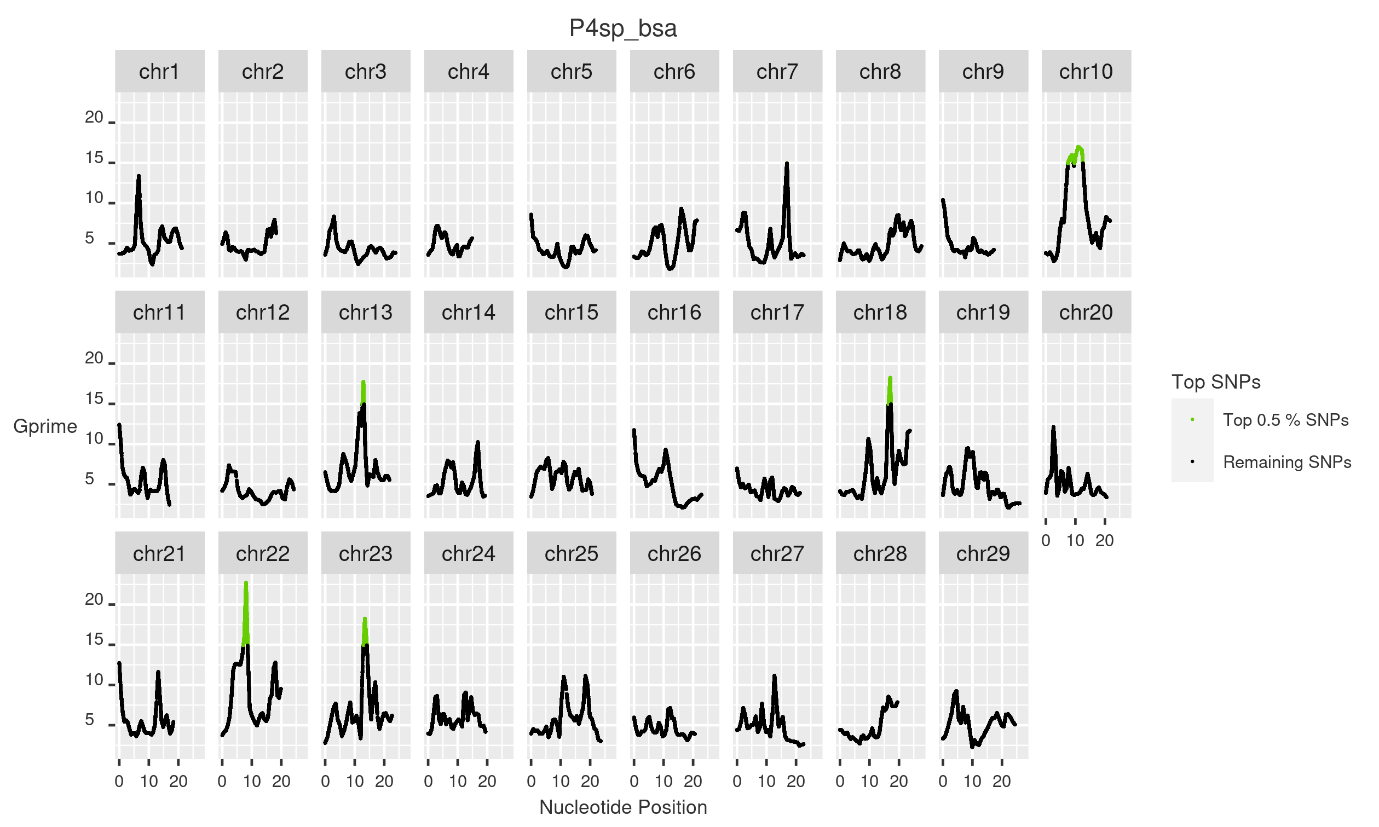


Bulked segregant analysis, B4, between sample bulk P4 and a bulk of parental WGS data contributing to P4.

Each analysed SNP site presents as a Gprime value analysed in a 1-Mb sliding window. Higher Gprime values reflect a higher frequency of P1 bulk alleles at certain genome positions than the bulk of parental WGS data. Green points represent the top 0.5% of Gprime values, with the remaining points coloured black. Six QTL peaks were found in B4: on Chromosome 7 at 16.91 Mb, on Chromosome 10 at 10.0 Mb, on Chromosome 13 at 12.95 Mb, on Chromosome 18 at 16.45 Mb, on Chromosome 22 at 7.17 Mb, and on Chromosome 23 at 12.97 Mb. The significance threshold was not presented for this bulk comparison as it was much higher than the top 0.5% of SNPs.


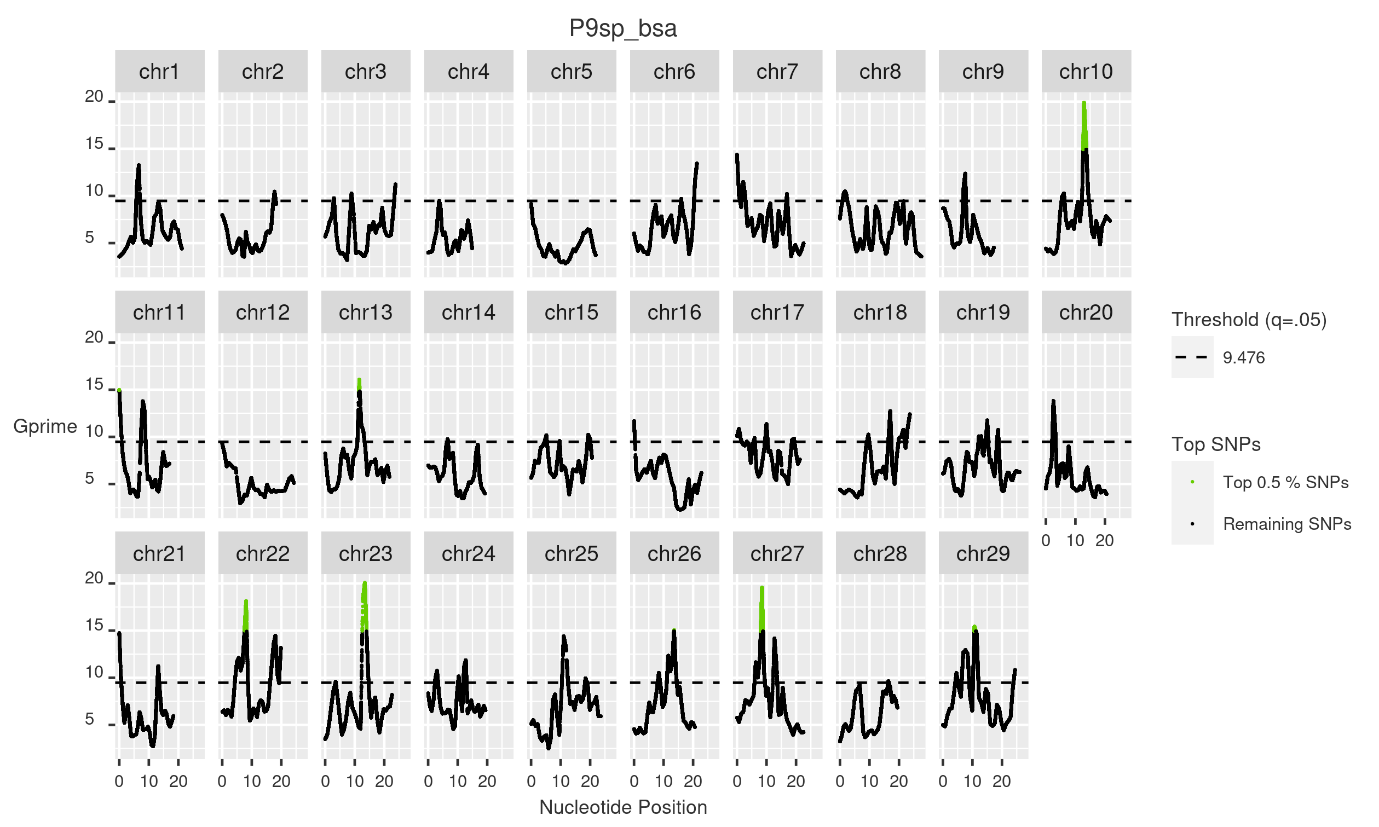


Bulked segregant analysis, B9, between sample bulk P9 and a bulk of parental WGS data contributing to P9.

Each analysed SNP site presents as a Gprime value analysed in a 1-Mb sliding window. Higher Gprime values reflect a higher frequency of P1 bulk alleles at certain genome positions than the bulk of parental WGS data. Green points represent the top 0.5% of Gprime values with the remaining points coloured black. The threshold of adjusted p = 0.05 is shown as a dashed line. Eight QTL peaks were found in B9: on Chromosome 10 at 12.61 Mb, on Chromosome 11 at 0.01 Mb, on Chromosome 13 at 11.55 Mb, on Chromosome 22 at 7.53 Mb, on Chromosome 23 at 12.56 Mb, on Chromosome 26 at 13.52 Mb, on Chromosome 27 at 8.02 Mb, and on Chromosome 29 at 10.64 Mb. The significance threshold was much lower than the top 0.5% of SNPs.


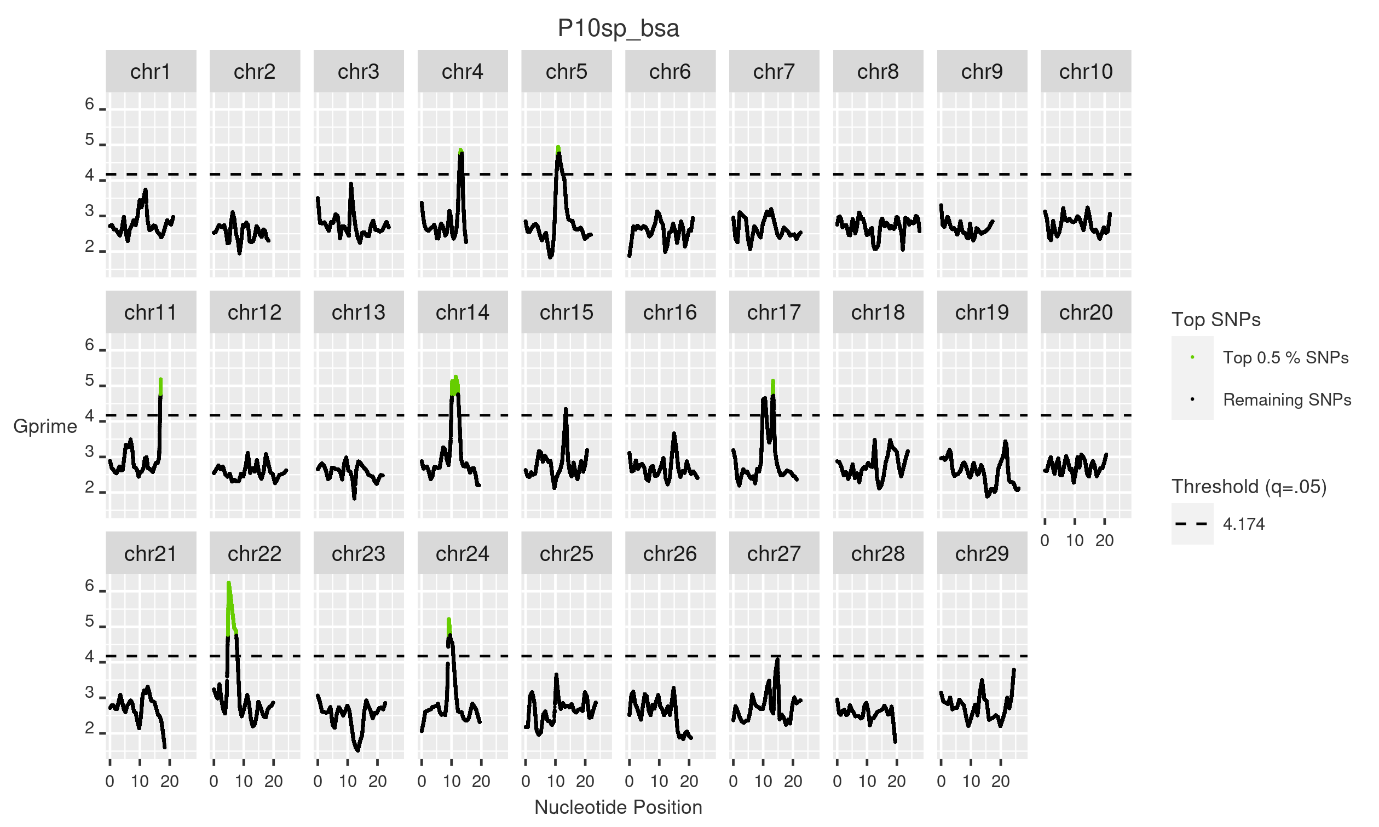


Bulked segregant analysis, B10, between sample bulk P10 and a bulk of parental WGS data contributing to P10.

Each analysed SNP site presents as a Gprime value analysed in a 1-Mb sliding window. Higher Gprime values reflect a higher frequency of P1 bulk alleles at certain genome positions than the bulk of parental WGS data. Green points represent the top 0.5% of Gprime values, with the remaining points coloured black. The threshold of adjusted p = 0.05 is shown as a dashed line. Seven QTL peaks were found in B10: on Chromosome 4 at 12.96 Mb, on Chromosome 5 at 10.81 Mb, Chromosome 11 at 16.90 Mb, on Chromosome 14 at 10.11 Mb, on Chromosome 17 at 13.15 Mb, on Chromosome 22 at 4.67 Mb, and on Chromosome 24 at 8.99 Mb. The significance threshold was lower than the top 0.5% of SNPs.


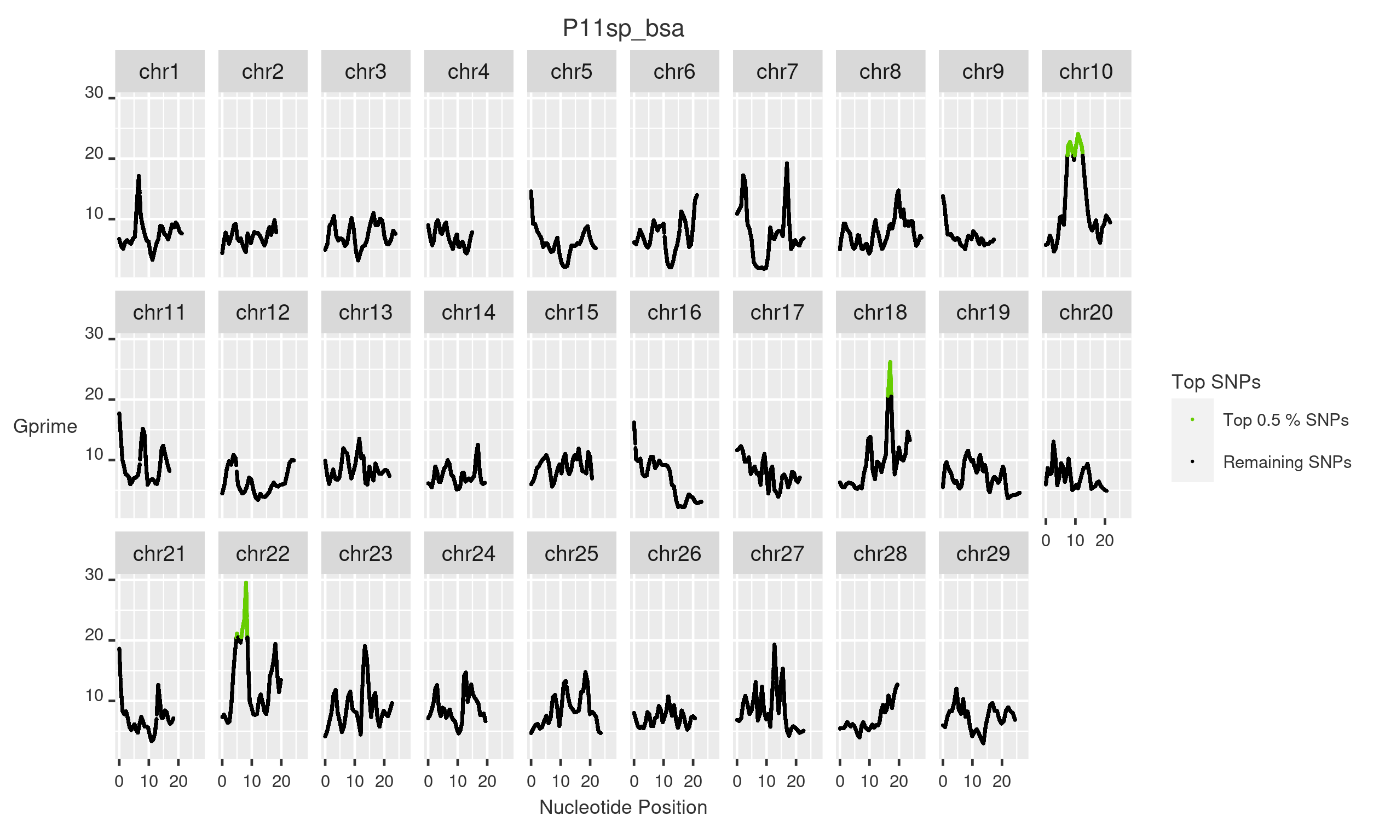


Bulked segregant analysis, B11, between sample bulk P11 and a bulk of parental WGS data contributing to P11.

Each analysed SNP site presents as a Gprime value analysed in a 1-Mb sliding window. Higher Gprime values reflect a higher frequency of P1 bulk alleles at certain genome positions than the bulk of parental WGS data. Green points represent the top 0.5% of Gprime values, with the remaining points coloured black. Three QTL peaks were found in B11: on Chromosome 10 at 10.0 Mb, on Chromosome 18 at 16.27 Mb, and Chromosome 22 at 4.84 Mb. The threshold of significance was not presented in this bulk comparison as it was much higher than the top 0.5% of SNPs.


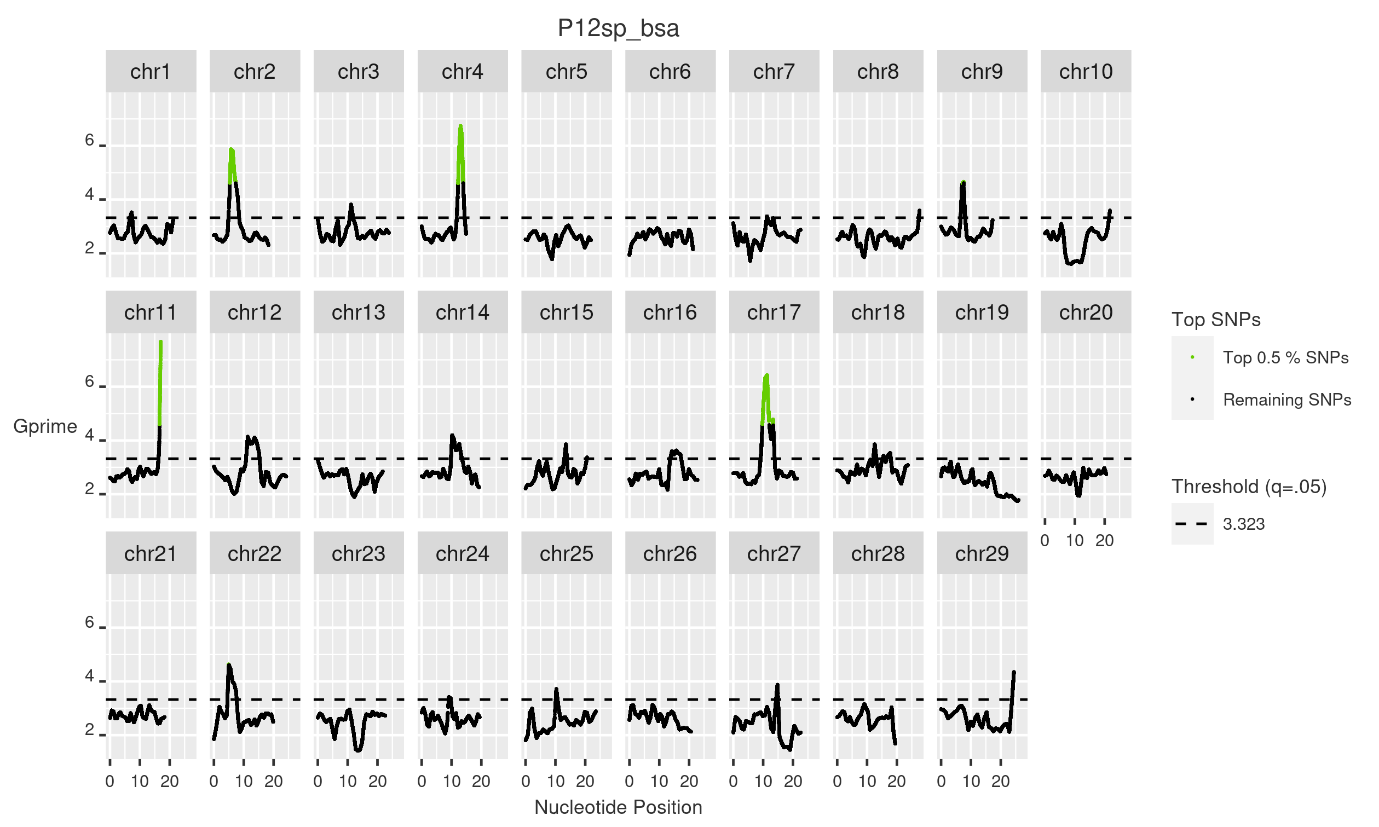


Bulked segregant analysis, B12, between sample bulk P12 and a bulk of parental WGS data contributing to P12.

Each SNP site presents as a Gprime value analysed in a 1-Mb sliding window. Higher Gprime values reflect a higher frequency of P1 bulk alleles at certain genome positions than the bulk of parental WGS data. Green points represent the top 0.5% of Gprime values, with the remaining points coloured black. The threshold of adjusted p = 0.05 is shown as a dashed line. Six QTL peaks were found in B12: on Chromosome 2 at 5.35 Mb, on Chromosome 4 at 12.22 Mb, on Chromosome 9 at 7.31 Mb, on Chromosome 11 at 16.60 Mb, on Chromosome 17 at 10.00 Mb, and on Chromosome 22 at 4.97 Mb. The significance threshold was much lower than the top 0.5% of SNPs.
